# Functional Outcome of Joshi’s External Stabilization System Fixation in Distal Radius Fractures

**DOI:** 10.7759/cureus.24215

**Published:** 2022-04-17

**Authors:** George Michael, Kitty George, Mathew A Canjirathinkal, Pranuthi Ratna, Jose Francis

**Affiliations:** 1 Orthopedic Surgery, Government Medical College Thrissur, Thrissur, IND; 2 General Practice, MES Medical College, Perinthalmanna, IND; 3 Radiodiagnosis, MD Anderson Cancer Center, Houston, USA; 4 Neurology, Kamineni Academy of Medical Science and Research Centre, Hyderabad, IND

**Keywords:** colles’ fracture, green and o’brien scoring, distal end radius, joshi’s external stabilization system, external fixator, wrist injury, radius fracture

## Abstract

Background

Distal radius fractures account for almost one-sixth of all fractures in a casualty setting. The usual aim of distal radius fracture treatment is to restore the function of the wrist joint, of which the distal radius is an important part. There seems to be no consensus regarding which mode of treatment is optimal for managing distal radius fracture, particularly when it is associated with distal radioulnar joint instability.

Objective

To describe the functional outcome in patients presenting with displaced distal radius fractures who undergo Joshi’s external stabilization system (JESS) fixation.

Methods

An observational study was done among 32 working-age (18 to 55 years) patients presenting with unilateral displaced distal radius fractures (excluding volar displaced) and subsequently treated with JESS fixation. The outcomes of the patients were assessed using the Green and O’Brien Scoring System modified by Cooney et al. at six months and one year following the surgery. Radiographs were also taken postoperatively and during follow-up. The data were analyzed (using IBM SPSS software version 22 and Microsoft Excel) in terms of the proportion of patients with acceptable clinical and radiological outcomes.

Results

Acceptable functional outcomes (good and excellent scores in the Green and O’Brien Scoring System) were observed in 78.1% of the study population. Though the functional outcome scores were higher among the younger age group, a statistically significant difference was not obtained. 96.9% of the patients had acceptable radiological reductions, and infection of the pin tracts complicated 9.4% of the cases. A significant improvement in outcome scores (p-value 0.0001) was observed between the outcome scores at six months and one year after surgery.

Conclusions

JESS fixation is an easy and effective method for treating displaced distal radius fractures to achieve good to excellent clinical outcomes. The functional outcome scores were better in the younger age group and male patients, but no statistically significant difference was observed.

## Introduction

Fracture as a disease contributes to a significant deal of morbidity worldwide and is being increasingly identified as a modern epidemic. A fracture occurs when more load is imparted to a bone than it can sustain. Osteoporotic bone can break with even a minor impact. Among all the fractures, the distal radius fractures are the commonest. It has been estimated that one-sixth of the fractures encountered in a casualty setting are distal radius fractures [1]. There is a bimodal age distribution for these fractures, with the first peak among young adults and the second among the elderly. The fracture mechanism can be as trivial as a simple falling on an outstretched hand, especially in the older age group. In younger patients, considerably more energy is required to cause fractures like in sports injuries and motorcycle accidents [2]. Worldwide, the incidence of distal radius fracture is increasing, especially among the younger age group.

The distal radius can be considered the most important bone of the wrist joint. Fractures of the distal radius will profoundly influence the movements of the wrist joint. Therefore, prompt treatment is required to restore the function of the wrist joint. The outcome of the distal radius fractures can reliably be predicted with the radiographically acceptable reduction of the distal radioulnar joint (DRUJ), which corresponds to anatomical reduction. Anatomical reduction is especially significant for the younger working-age group. For example, a 23-year-old athlete may want to resume competition, but an 80-year-old person usually only wants to return to activities of daily living. Therefore, treatment goals must be tailor-made to each patient. However, treatment should be determined not by age but by activity level [3].

Treatment of distal radius fractures widely varies according to the treatment goals, ranging from cast immobilization to operative management. Although a wide variety of management options are available for distal radius fractures, there seems to be no consensus regarding the optimal therapy [4]. In the study “Cast Immobilisation vs. Wire Fixation in the Management of Middle-aged and Elderly Patients with Distal Radius Fractures,” Jordan et al. find that cast immobilization can lead to poor radiological outcomes, but the functional results are comparable to operative management in the elderly [5]. Still, poor radiological outcomes can lead to poor functional results, especially in the younger age group. Since a better radiologically acceptable fracture reduction is attained using operative methods, there is an increase in the popularity of the surgical fixation of distal radius fractures. Sander et al. suggest that the indications for surgery should correlate with the fracture’s severity, not age [6].

Displaced fractures not reducible with closed manipulation are preferably treated by surgery. Operative treatment methods include percutaneous pinning, external fixator, and open reduction with internal fixation. The number of people undergoing surgical fixation increases due to better outcomes associated with the anatomical reduction of the DRUJ and wrist joint. External fixators are used for the management of fractures to obtain and maintain reduction throughout the treatment period, usually without touching the fracture region. Joshi’s external stabilization system (JESS) is an external fixator developed by Dr. B. B. Joshi et al. from Mumbai. Even though JESS is weaker than an Ilizarov fixator [7] because it is cost-effective and easily applicable, it can be used for various conditions, including burn scar contracture and clubfoot [8,9], and has a prominent place in managing displaced distal radius fractures.

External fixation is the technique of skeletal immobilization by fixing percutaneous pins into the bones and linking them to connecting rods placed external to the body, forming a rigid framework. JESS is an external fixator that works on the principle of ligamentotaxis, which uses continuous longitudinal force or distraction to bring fracture fragments to close together. The longitudinal distraction force causes the soft tissues surrounding the fracture to help mold the bony fragments and facilitate reduction. JESS is a simple, cost-effective technique with a minimal learning curve to master its application and can be an excellent alternative to cast immobilization, especially when good radiological outcomes are warranted.

The functional outcome after distal radius fractures can be measured using several scoring systems, including the widely used Green and O’Brien Scoring System [10]. This system was subsequently modified by Cooney et al. which has four headings: pain, occupation, range of motion, and grip strength. If the hand is injured, then dorsiflexion-palmar flexion arc is used for scoring instead of grip strength. The evaluation of function can be done during the follow-up periods after the procedure.

## Materials and methods

Study design and population

This was a prospective observational study involving patients presenting with fractures of the distal radius and treated with JESS at our hospital from February 2017 to January 2018. The inclusion criterion was fractures of the distal end of the radius limited to the age group 18 to 55 years, the group most probable to benefit best by intervention. The exclusion criteria were undisplaced fractures of the distal end of the radius where the JESS is not indicated, fractures with volar displacement in which plating is indicated, and bilateral fractures of the distal radius where the wrist function cannot be compared against the unaffected side. For more straightforward data analysis, the patients were further subdivided into less than or equal to 40 and greater than 40 years of age.

Sample size

The sample size was calculated by the following formula:

$n= \frac{z_{a}^{2}pq}{d^{2}}$

Where n = sample size,

Z_a_ = 1.96,

p = the percentage of patients with acceptable (good or excellent Green and O’Brien score) outcomes in the study “A multifactorial study of application of Joshi’s External Stabilizing System in Displaced Distal End Radius Fractures” by Shukla et al. published in the Indian Journal of Basic and Applied Medical Research, December 2013 [11],

q = 100 − p,

d = maximum allowable error (20% of p)

i.e., $n= \frac{1.96^{2}*77.8*22.2}{15.56^{2}}$ = 27.4

Thus it was estimated that a sample size of 28 patients is deemed satisfactory for describing the functional outcome.

Joshi’s external stabilization system

**Figure 1:**
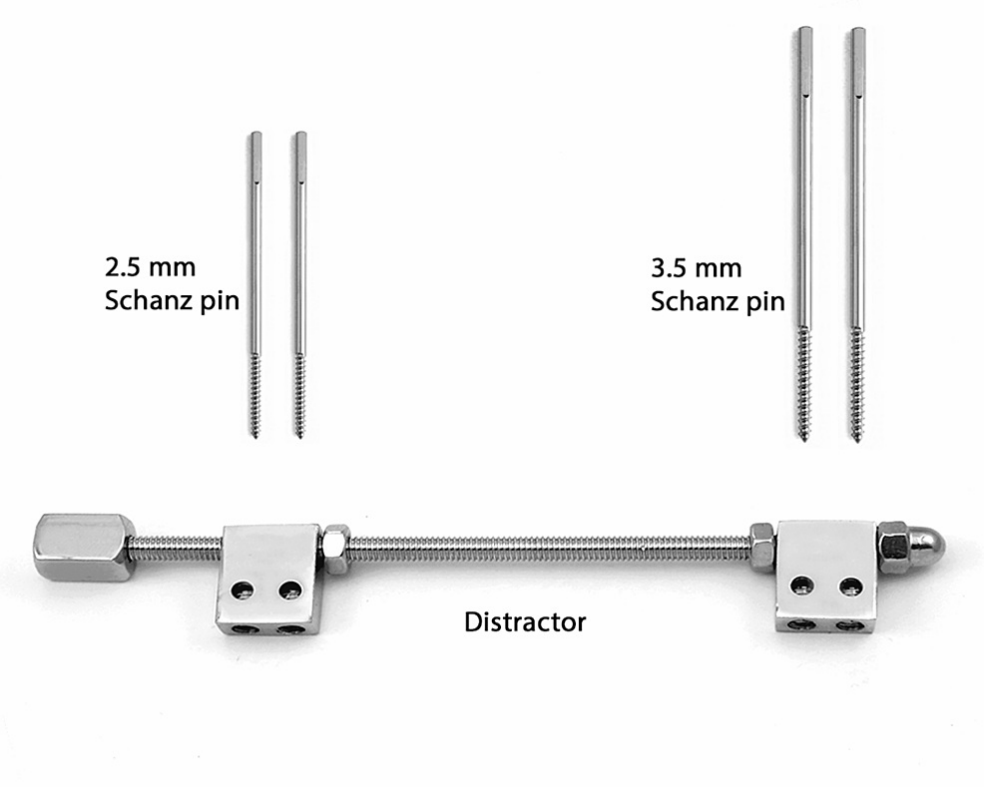
Parts of Joshi’s external stabilization system

JESS [12] is an external fixation system based on the principles of ligamentotaxis. When used for the management of distal radius fractures, JESS consists of applying four pins in the radius and the second metacarpal connected by a threaded or serrated connecting rod with provision for distraction. Two 3.5 mm Schanz pins are applied in radius 2 to 3 cm proximal to fracture, preferably under direct vision, to avoid injury to the superficial sensory branch of the radial nerve at the junction between the distal and middle third of the radius. The two 2.5 mm Schanz pins are applied in the proximal part of the second metacarpal’s bare area between the first dorsal interossei and the extensor tendons through the JESS distractor slots. The distractor is then used to attain reduction by ligamentotaxis. An image showing the components of JESS is demonstrated in Figure 1.

Clinical assessment of the wrist

Clinical examination still reigns supreme for functional evaluation despite advances in the imaging and technical modalities for assessing the wrist [13]. For examination, the hand, wrist, and forearm must be adequately exposed above the elbow level. Inspection is done to look for swellings, deformities, muscle wasting, scars, discoloration, engorged veins, and other skin/nail changes. Palpation is done to assess any local rise of temperature or tenderness. Any nodules, bony irregularity, thickened tendon, or nerve should be felt. Radial and ulnar pulsations should be tested (including Allen’s test).

The capillary filling test may be done to assess the status of the delicate vasculature of the hand. Sensations should be tested in the relevant areas supplied by the radial, median, and ulnar nerves. Movements of the wrist and grip strength can provide valuable insight into the function of the wrist. Special tests like Phalen’s and reverse Phalen’s tests also have a limited role in the assessment.

The outcome of treatment after JESS fixation was evaluated using the Green and O’Brien Scoring System, which assesses wrist function under four categories, namely pain, activity, range of motion, and grip strength. Each has a minimum score of 0 and a maximum of 25. The scores from these headers are summed together to yield the final score. The modified Green and O’Brien Scoring System used in the study is detailed in Table 1.

**Table 1:** Modified Green and O’Brien Scoring System

Score Category		Score
Pain		
	None	25
	Mild, occasional	20
	Moderate, tolerable	15
	Severe, intolerable	0
Occupation		
	Returned to regular employment	25
	Restricted employment	20
	Able to work but unemployed	15
	Unable to work because of pain	0
Range of Motion in Degrees		
	120 or more	25
	91to 119	15
	61to 90	10
	31 to 60	5
	30 or less	0
Grip Strength		
	Full	25
	75% to 99% of normal	15
	50% to 74% of normal	10
	25% to 49% of normal	5
	24% or less	0
Total Score		
	Excellent	90 or more
	Good	80 to 89
	Fair	65 to 79
	Poor	64 or less

Study procedure

Written informed consent was taken from all subjects. A questionnaire containing questions like age, gender, side of the fracture, comorbidities, and complications was used. Sociodemographic details were recorded. For the indicated cases, a JESS fixator was applied after preoperative radiographs, and follow-up radiographs were taken immediately after surgery. Criteria for acceptable reduction based on already established guidelines for the age group under study included radial inclination greater than 15o, radial length with less than 5 mm shortening, radial tilt with less than 5o dorsal tilt, and articular incongruity with less than 2 mm step-off [14]. IV antibiotics were given for the first three days and then for one-week oral antibiotics. Finger mobilization was initiated from the first post-op day. Pin tract care was advised comprising daily showers and dry dressings. At four weeks, in stable cases, JESS was removed, and a short arm cast was applied. Casts were removed at six weeks and reviewed at six months and one year, along with fresh X-rays. In unstable cases, JESS was to be retained till six weeks, but no case in the study required retainment of JESS till six weeks.

Outcomes of patients were assessed using the Green and O’Brien Scoring System modified by Cooney et al. at six months and one year following surgery. The final score of 80-89 is considered as good result, and 90-100 is regarded as an excellent result, both of which are acceptable outcome results. Data were segregated and analyzed using Microsoft Excel and SPSS (IBM, Armonk, New York, USA) software version 22. Two-tailed t-test assuming unequal variance was performed to compare two age groups (<=40 years vs. > 40 years) and two gender groups (males vs. females).

## Results

All the patients in this study were treated on an outpatient basis. On average, the procedure of the JESS application took about 25 minutes. The JESS distractor was maintained for four weeks, and further immobilization in the cast was done for two more weeks. Among the 32 patients who were studied, the mean age was 39.8 years, with a standard deviation of 10.06. Most patients were older than 40 years, and in all the age groups, around two-thirds were males. All the patients in the study were right-handed, and nearly two-thirds of the fractures were on the right side. As per the previously set exclusion criteria, patients with bilateral distal radius fractures were not included in the study to ensure that the functional outcome scores were measured objectively by comparing the function to the unaffected limb.

As expected, high energy trauma like road traffic accidents was the primary cause of the fracture in this study because the patient group belongs to the younger working-age group. Traumatic causes like falling from a standing height on an outstretched hand are classified as low-impact injuries. Low energy impact injury is the most common cause of distal radius fractures in the general population because these fractures are commonly seen in the osteoporotic elderly. In the strong bones of younger, a considerable force is required to cause distal radius fractures.

A radiologically acceptable reduction was not attained in one patient with suboptimal functional outcome scores at six months and one year. However, sufficient data were not available to conclusively identify the correlation between radiologic reduction and acceptable functional outcomes.

Three patients (9.38%) had diabetes mellitus. All three had reasonable glycemic control and were on medications. Five (15.63%) patients (including one with diabetes) had hypertension. No other comorbidities were recorded in the study population. Three patients (9.38%) developed pin tract infection after the JESS fixation. Of these, one patient had diabetes mellitus, and one had hypertension. All cases of infection had closed fractures, and no infection was reported among the open fracture group.

Reduction of pain is the most crucial aspect as far as the patient is concerned. Returning to previous activity is also an essential part of the functional outcome because it objectively measures morbidity reduction following the treatment. After the procedure, the patients were followed up at six months and one year to measure the functional outcome based on the Green and O’Brien score, and the JESS was found to be helpful in the mitigation of pain in distal radius fractures allowing resumption of prior activity. All the patients had a poor range of motion after the procedure, probably because of stiffness of the wrist joint caused by prolonged immobilization. The grip strength was measured objectively by comparing it with the unaffected opposite side. The results were much better at the end of one year when compared to six months. Outcomes were much more favorable in those less than 40 years, with just one patient having a low functional score. The mean of the functional outcome scores in males was better than in females. The distribution of functional outcome scores is detailed in Table 2.

**Table 2:** Distribution of functional outcome scores

Score Category	Mean ± standard deviation	
	6 months	1 year
Pain	19.8 ± 3.2	23.4 ± 2.4
Occupation	21.9 ± 4.7	23.9 ± 2.5
Range of motion	11.7 ± 3.7	15.3 ± 4.2
Grip strength	17.5 ± 6.5	21.1 ± 5.6
Total score	70.9 ± 14.6	83.8 ± 10.0

Two-tailed t-test assuming unequal variance was performed to compare two age groups (<=40 years vs. > 40 years) and two gender groups (males vs. females). The p-value for the age group comparisons was 0.2129, and that for gender comparisons was 0.1679. The tests failed to reject the null hypothesis (H_o_). Therefore, the difference between the age and gender groups was not statistically significant. However, all groups showed statistically significant improvement in the outcome score at one year compared to the six-month levels (p-value 0.0001). Table 3 describes the statistical comparison of the Green and O’Brien scores at six months and one year after the procedure.

**Table 3:** Comparison of functional outcome scores at six months and one year

Score Category	p-value	
Pain	0.000004	
Occupation	0.034344	
Range of motion	0.000593	
Grip strength	0.021069	
Total score	0.000121	

## Discussion

Anatomy of the distal radius

The radius is one of the two bones of the forearm and lies on the lateral side. It is a long bone with a head and neck proximally, a slightly curved shaft, and a broader distal end. The shaft of the radius is triangular in cross-section throughout most of its length with three borders (anterior, posterior, and interosseous) and three surfaces (anterior, posterior, and lateral). The interosseous border is sharp and serves as the attachment site for the interosseous membrane, which links the radius to the ulna. However, distally the radius is quadrilateral and has two articular surfaces-one below for the carpal bones and another medially for the ulna [15].

The articular surface of the distal end of the radius is distinct, with two facets for the carpal bones-scaphoid (triangular and situated laterally) and lunate (quadrangular and medially located). The medial surface at the distal end of the radius has a facet for articulating with the ulna, called the ulnar notch (sigmoid cavity). The carpal and ulnar articular surfaces are separated by a prominent ridge to which the base of the triangular articular disk is attached; this disk separates the DRUJ from the wrist joint.

The distal radius has expansive anterior and posterior surfaces compared to the narrow medial and lateral surfaces. The posterior surface of the distal end of the radius is characterized by a dorsal tubercle, which acts as a pulley for the extensor pollicis longus tendon. The lateral surface of the radius is diamond-shaped and extends distally to form the radial styloid process. The cortical bone is thinner on the posterior and lateral surfaces making the fractures collapse dorso-radially. Attachments to the distal radius include the brachioradialis muscle that inserts just above the styloid process, the pronator quadratus muscle inserted into the anterior surface, the extensor retinaculum, the interosseous membrane, the articular capsule of the wrist joint, and the articular disk.

The distal radius is also connected to the carpal bones and ulna by a series of ligaments. The palmar radiocarpal ligament complex consists of the radioscaphocapitate, long radiolunate, radioscapholunate, and short radiolunate ligaments. The ulnocarpal ligaments include the ulnolunate, ulnotriquetral, and ulnocapitate ligaments. Rather than attaching directly to the ulna, the ulnocarpal ligaments attach to the palmar radioulnar ligament. The dorsal radiocarpal ligament spans the radiocarpal joint on the dorsal side. The DRUJ is connected by the triangular fibrocartilage complex (TFCC), which is formed by the fibrocartilaginous disk and the palmar and dorsal radioulnar ligaments. Therefore, these ligaments are at an inevitable risk of being injured in the distal radius fractures.

Biomechanics and classification

There is a three-column concept backed by using pressure sensors in cadaveric wrists under unphysiological conditions and in healthy volunteers under physiologic conditions. Under this theory, the wrist has three columns for load transmission: radial, intermediate, and ulnar columns [16,17]. The radial column is composed of the radial styloid with the scaphoid facet. The intermediate column consists of the lunate facet and the sigmoid notch. The ulnar column is formed by the ulnar head, the DRUJ, the ulnar styloid, and the TFCC.

Smaller loads are transmitted via the radial column. Its functions include serving as a stabilizer, as a bony buttress radially (which limits radial deviation), and as an insertion for the ligaments (which limits ulnar deviation by a tension band mechanism). The intermediate column serves primarily for axial load transmission from the lunate and the proximal pole of the scaphoid. About half of the load is transmitted across the triangular fibrocartilage and the ulnar column, which is also the stabilizing pivot of the wrist. The radius turns around this pivot of the ulna.

The understanding of biomechanics associated with the distal radius profoundly influences open reduction and internal fixation and serves as a baseline for the classification systems based on the mechanism of injury. All types of distal radius fractures can be reproduced by hyperextension of the wrist, which is the single most important mechanism of distal radius fracture. Even though several classifications are available for distal radius fractures, one that is routinely used is the patho-mechanical classification system of Fernandez based on the forces that act upon the wrist at the moment of the impact [18]. This classification is also used to guide the treatment of distal radius fractures. Other classification systems are less helpful than the Fernandez system in dictating the mode of treatment. Frykman classification is based on the joint involvement (radiocarpal and radioulnar) with or without the ulnar styloid fracture. Melone divides intraarticular fractures based on displacement into five types. The Arbeitsgemeinschaft für Osteosynthesefragen (AO) classification is more comprehensive but cumbersome to use. Several other classifications are also available, and their discussion is beyond the scope of this study.

Investigations

A CT scan is helpful for intraarticular fractures, particularly for preoperative planning. MRI is useful in evaluating the soft-tissue injuries associated with 70% of the distal radius fractures, including TFCC injuries, scapholunate ligament injuries, and lunotriquetral ligament injuries. However, as in the case of fractures in general, when a patient presents with a history of distal radius fracture, the initial investigation ordered is the plain radiographs of the wrist in two planes (anteroposterior and lateral) [19]. Based on the radiograph, the fracture is classified, and appropriate treatment is decided considering the patient-specific needs. The normal radiological anatomy is to be understood to know the extent of the success of intervention as well.

Treatment and complications

**Figure 2:**
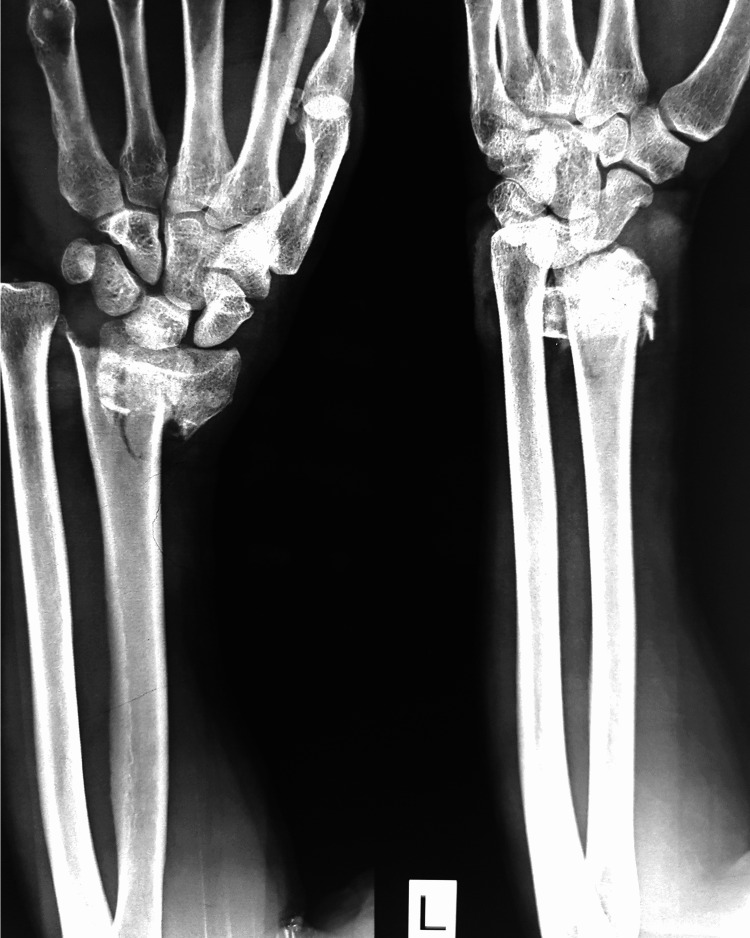
X-ray of a distal radius fracture before management

The goal of fracture treatment is to restore functionality, and this depends on the patient’s prior activity level. A successful outcome is correlated with the accuracy of anatomic reduction, restoration of anatomic relations, and early efforts to regain movement of the wrist and fingers. The methods of treatment are broadly divided into non-operative and operative. Closed reduction with cast immobilization is the most commonly administered form of treatment for distal radius fractures, particularly in osteoporotic fractures in the elderly. Indications for this conservative therapy include extraarticular fracture, less than 5 mm radial shortening, and dorsal angulation less than 5 degrees or within 20 degrees of the contralateral distal radius after a closed reduction. One troublesome complication to the conservative management is the rupture of the extensor pollicis longus tendon, most probably due to ischemia. It can also lead to acute carpal tunnel syndrome. An X-ray image of a patient who suffered from a closed comminuted distal end of radius fracture is shown in Figure 2.

Operative techniques for treating distal radius include closed reduction and percutaneous pinning (CRPP), external fixation, and open reduction and internal fixation (ORIF). Operative treatment is best suited for unstable distal radius fractures where conservative treatment would most likely fail. The outcomes for the CRPP procedure are excellent, provided proper case selection is first employed. An essential prerequisite for its success is the presence of a stable volar cortex. Techniques include the Kapandji intrafocal technique and the Rayhack technique with arthroscopically assisted reduction. ORIF utilizes plates and screws to help maintain reduction. Volar plating is preferred over dorsal plating. It can be combined with pinning and external fixation as well. Tendon irritation due to the implant is a possible complication.

**Figure 3:**
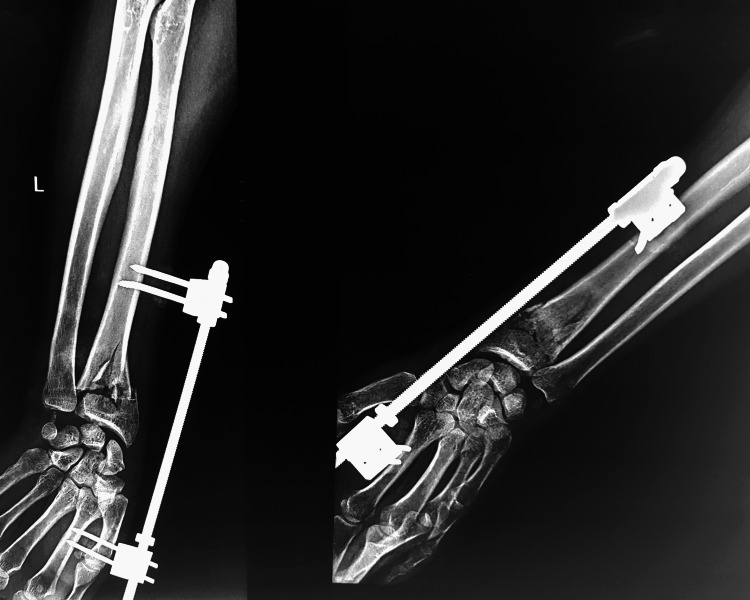
Distal radius fracture after JESS fixation JESS: Joshi’s External Stabilization System

External fixation relies on ligamentotaxis for maintaining reduction. One significant drawback is the inability to restore palmar tilt. Therefore, external fixation is frequently combined with other procedures like pinning to mitigate this restriction. External fixation is especially useful in an open fracture where regular wound care needs to be given. There are two types of external fixation based on whether the fixator crosses/spans the wrist joint or not. Non-spanning external fixation is useful when the distal fragment is large enough to be pinned and connected to the fixator framework. Spanning external fixation crosses the wrist joint, does not fix the distal fragment, and relies solely on ligamentotaxis for reduction. When used in distal radius fractures, JESS is a form of spanning external fixator connecting the proximal fragment of the fractured radius and the second metacarpal crossing the wrist joint. In addition to the complications inherent to all distal end of radius fractures such as malunion, nonunion, and stiffness, the external fixators can also cause pin-related complications like infection and iatrogenic injury to the superficial branch of the radial nerve. Post-procedure radiological imaging can help adjust the amount of distraction applied at the fracture site. A postoperative X-ray image from the aforementioned patient immediately after JESS fixation is shown in Figure 3.

Functional outcome in the study group

The most common fracture mechanism in the study group was a road traffic accident rather than a simple fall on an outstretched hand, probably because the strong bones of the younger adults required considerable force for the fracture to occur. Most of the patients in the study were free of comorbidities because, in our institution, JESS is generally used in patients without any associated significant illness. Radiologically acceptable reductions were achieved in all but one patient (about 97%). Due to data constraints, the association between the outcome and reduction could not be assessed. Three patients (9.4%) developed pin tract infection following the surgery and were treated successfully with antibiotics.

The functional outcome scores following JESS fixation were measured at six months and one year after the procedure during the follow-up visits using the Green and O’Brien scoring system. The mean outcome scores were 70.9 ± 14.6 at six months and 83.8 ± 10.0 at one year. A score of 80 or more is considered acceptable. Around 40.6% of the patients had acceptable functional outcome scores (i.e., more than 80) at six months following surgery which grew to 78.1% by one year. The outcome scores were also better in the younger males, although no statistically significant difference was perceived. The findings were comparable to similar studies undertaken previously [11,20,21,22] and are shown in Table 4.

**Table 4:** Similar studies

Study	Mean Age	Acceptable Functional Outcome
Jupiter et al. (1996) [20]	42 years	83.60%
Kapoor et al. (2000) [21]	39 years	80%
Shukla et al. (2013) [11]	40 years	77.80%
Chaudhuri et al. (2014) [22]	43 years	66%
Present study	40 years	78.10%

The fractures of the distal radius are quite common, and various treatment options are available to manage them. External fixators are invaluable in the case of open fractures because better wound care can be provided. The lack of internal implants also makes the post-surgical infection less worrisome. One significant difficulty faced after continuous immobilization is the stiffness of joints which has significantly affected the functional outcome scores. Prolonged and intense physiotherapy may be required in such cases for recovery, as evidenced by the improvement in the range of motion scores at one year after surgery. Newer techniques like dynamic external fixators show much promise in the early mobilization that may lead to better functional outcomes due to the decreased chance of joint stiffness. However, the choice of management should be patient-centric, which takes the patient’s requirements to the center stage. There is no need for aggressive strategies in an older adult whose functional needs will be much less than a young and active adult.

Study limitations

Even though the current study showed acceptable outcomes with JESS fixation, there were no definite conclusions regarding the relationship between anatomic/radiologic reduction and outcome, and a significant difference could not be observed between younger and older age groups. These limitations could be addressed with a study with more statistical power, possibly by using a larger sample size in the future. The study also lacked a proper comparison group, and the results should therefore be compared with other studies.

## Conclusions

JESS, a highly effective technique available for treating distal radius fractures, has an easy learning curve and cost-effectiveness, yielding an acceptable functional outcome in most patients with minimal complications and pitfalls. When properly applied to the indicated fracture patterns, it can result in a radiologically acceptable reduction in most cases. However, its effectiveness is limited to the fracture patterns to which it can be applied. Knowledge regarding classification systems aids in understanding whether ligamentotaxis can be used for a specific fracture pattern or not. Emphasis on early mobilization and physiotherapy can definitely enhance the functional outcome.
